# Supplementary material for: Aberrant cortical spine dynamics after concussive injury are reversed by integrated stress response inhibition
Source: Proc Natl Acad Sci U S A. 2022 Oct 13;119(42):e2209427119. doi: 10.1073/pnas.2209427119 (PMC9586300; doi:10.1073/pnas.2209427119)
Supplement: Supplementary File [file pnas.2209427119.sapp.pdf]

PNAS

SI Appendix for

Aberrant cortical spine dynamics after concussive injury are reversed by integrated stress response inhibition.

Elma S. Frias[1,2,10], Mahmood S. Hoseini[3], Karen Krukowski[1,2,10], Maria Serena Paladini[1,2,10], Katherine Grue[1,2,10], Gonzalo Ureta[4], Kira D.A. Rienecker[1,2], Peter Walter[5,6,10], Michael P. Stryker[3,8,9], and Susanna Rosi[1,2,7,8,9,10].

Corresponding Authors:
Susanna Rosi, Ph.D.
Altos Labs, Redwood City, CA 94065
Phone number: (415) 342-6244
Email: srosi@altoslabs.com

Michael Stryker, Ph.D.
675 Nelson Rising Lane, Room 535
Department of Physiology
University of California San Francisco
San Francisco, CA 94143
Phone number: (415) 502-7380
Email: michael.stryker@ucsf.edu

Peter Walter, Ph.D.
Altos Labs, Redwood City, CA 94065
Phone number: (415) 595-9770
Email: pwalter@altoslabs.com

This PDF file includes:

Figures S1 to S8

Tables S1 to S1

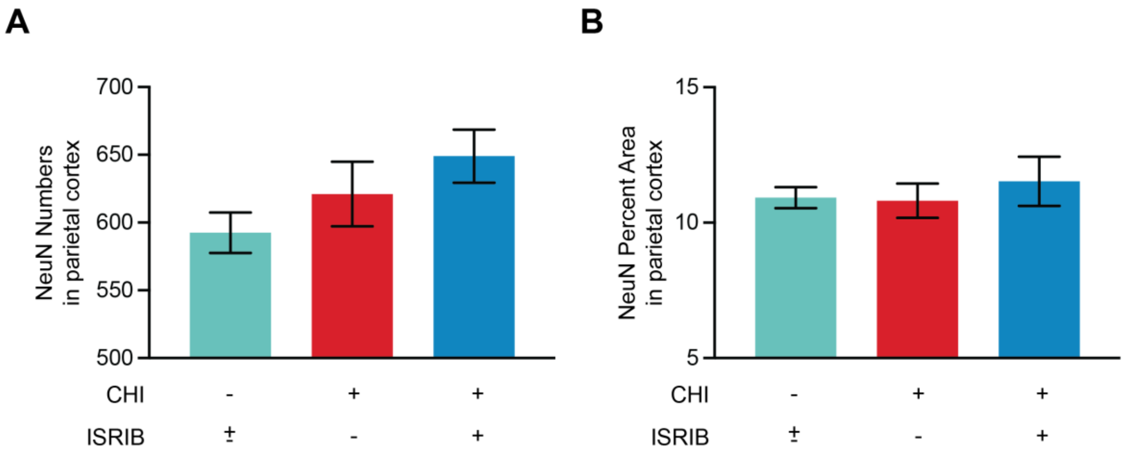

Supplemental Figure S1. No changes in neuronal numbers or percent area in the parietal cortex following closed-head injury or ISRIB treatment.

(A-B) Neuronal cell bodies were quantified in the parietal cortex from adult male WT mice. Neuronal nuclei protein NeuN was used to count neuronal cell bodies. No significant differences in NeuN numbers or percent area were found at 10x and 20x magnification. **Statistics:** Analysis was done by Ordinary one-way ANOVA followed by multiple comparisons using Tukey-post hoc (A-B). All data are means ± SEM. Sham ± ISRIB n = 5; CHI n = 4; CHI + ISRIB n = 4.

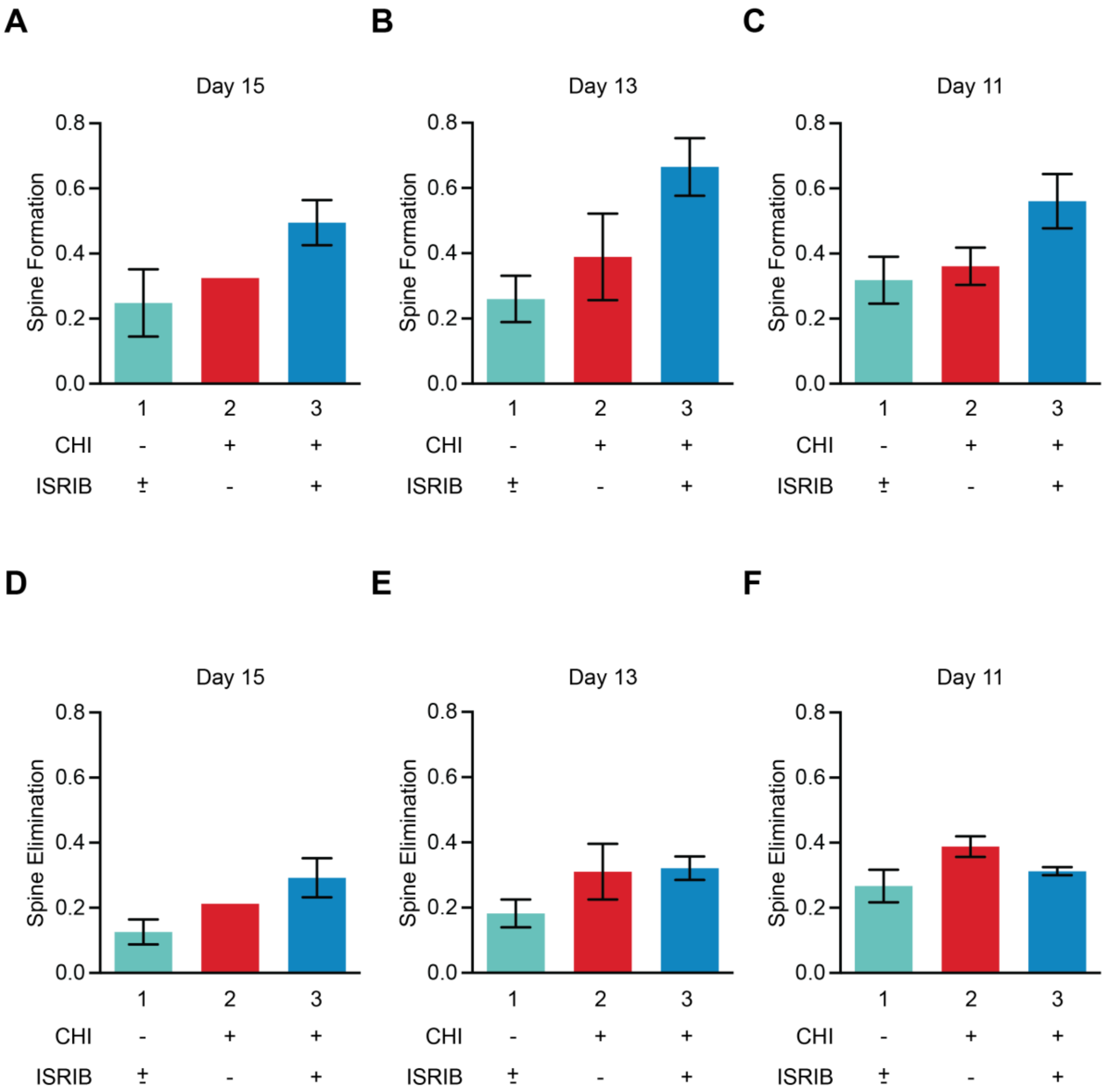

Supplemental Figure S2. Reverse analysis using day 18 as the baseline imaging day.

Reverse-time analysis of **(A-C)** spine formation and **(D-F)** elimination using day 18 as the baseline imaging day**.** All data are means ± SEM. Sham ± ISRIB n = 13; CHI n = 12; CHI + ISRIB n = 11.

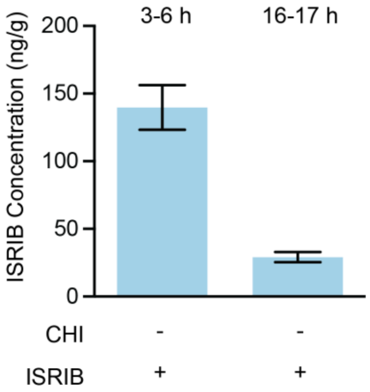

Supplemental Figure S3. ISRIB concentration in the brain.

ISRIB concentration (ng/g) in the brain of control WT mice at 3-6h or 16-17h post last injection. All data are means ± SEM. Sham ± ISRIB n = 12, 6, respectively.

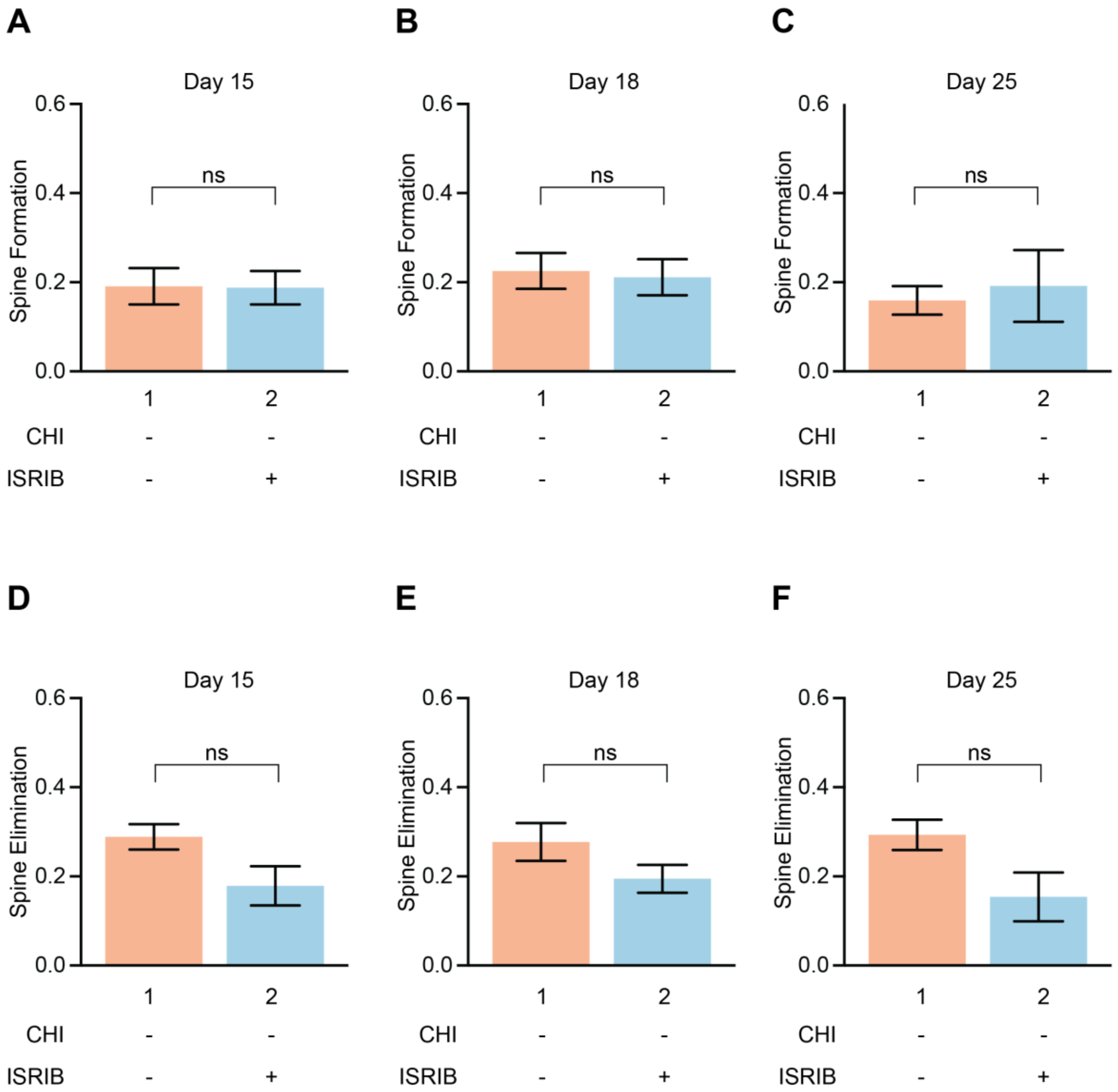

Supplemental Figure S4. ISRIB treatment does not affect spine formation or elimination of sham control mice.

(A-C) Spine formation and **(D-F)** spine elimination at days 15, 18, and 25 dpi for Sham – ISRIB (Bar 1) and Sham + ISRIB (Bar 2). ISRIB treatment had no effect on sham control mice. The number of animals in each group was n = 6 and 7, respectively. **Statistics:** Analysis was done by Unpaired T-test (A-F). All data are means ± SEM. Sham ± ISRIB n = 13.

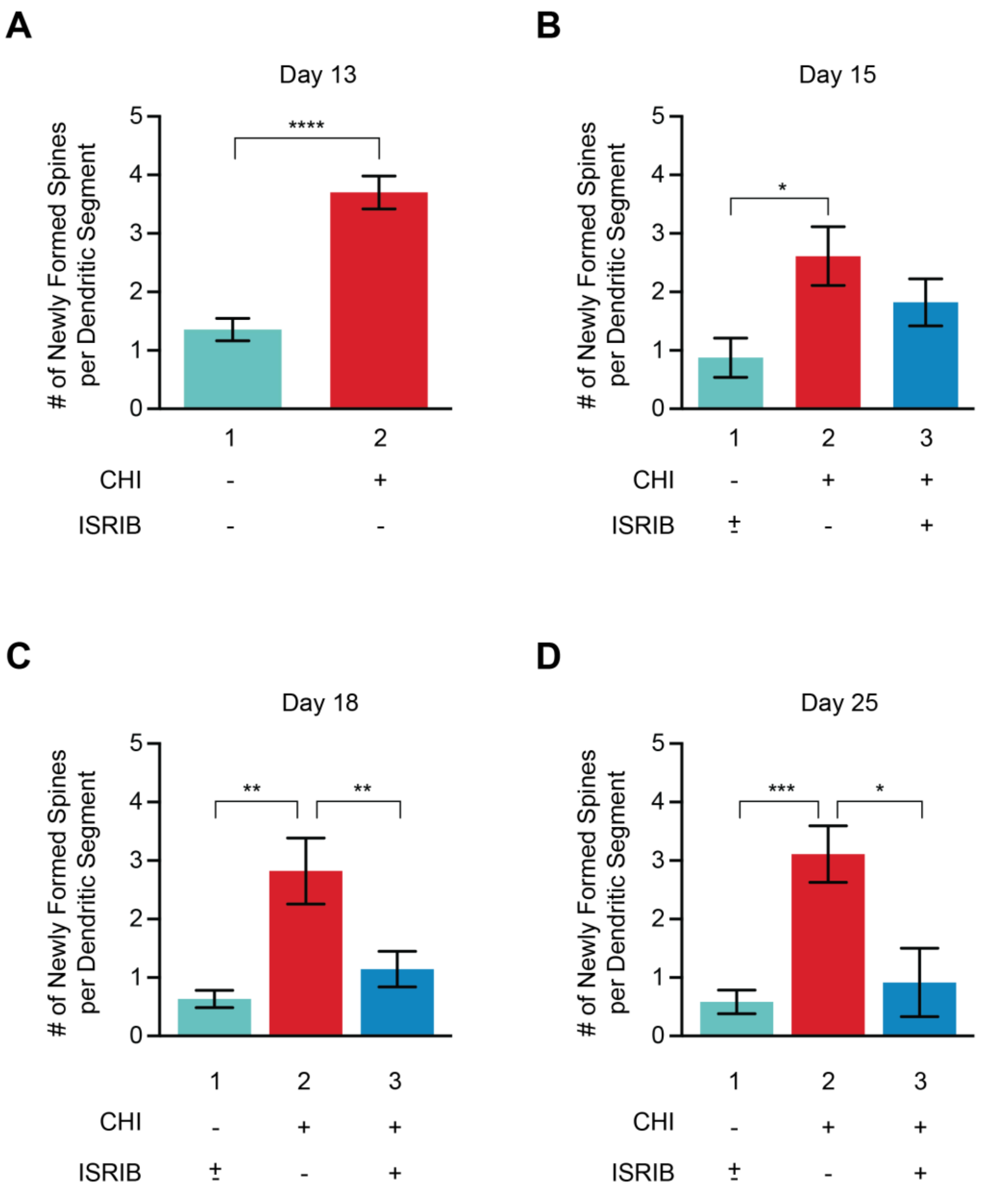

Supplemental Figure S5. Number of newly formed spines at days 13-25 post-injury.

(A-D) Number of newly formed spines in Sham ± ISRIB (Bar 1), CHI (Bar 2), and CHI + ISRIB (Bar 3) mice at days 13-25 post-injury (dpi). **Statistics:** Analysis was done by Unpaired T-test (A) or Ordinary one-way ANOVA followed by multiple comparisons using Tukey-post hoc (B-D). $p < 0.05$ (*), $p < 0.005$ (**), $p < 0.0005$ (***), $p < 0.0001$ (****), as indicated in the figure. All data are means ± SEM. Sham ± ISRIB n = 13; CHI n = 12; CHI + ISRIB n = 11.

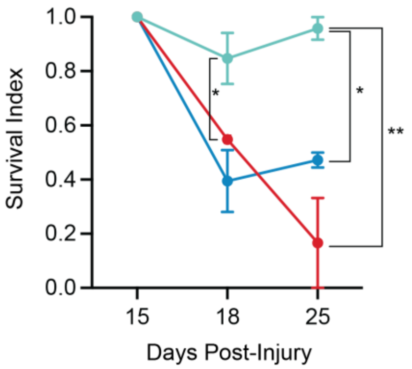

Supplemental Figure S6. Survival index of spines born between days 13 and 15 and indexed on days 18 and 25 at days 15-25 post-injury.

Survival index in Sham ± ISRIB (light blue line), CHI (red line), and CHI + ISRIB (dark blue line) mice at days 15-25 post-injury demonstrate same trends as seen in Figure 3C. **Statistics:** Analysis was done at each dpi by Ordinary one-way ANOVA followed by multiple comparisons using Tukey-post hoc. $p < 0.05$ (*), $p < 0.005$ (**), as indicated in the figure. All data are means ± SEM. Sham ± ISRIB n = 13; CHI n = 12; CHI + ISRIB n = 11.

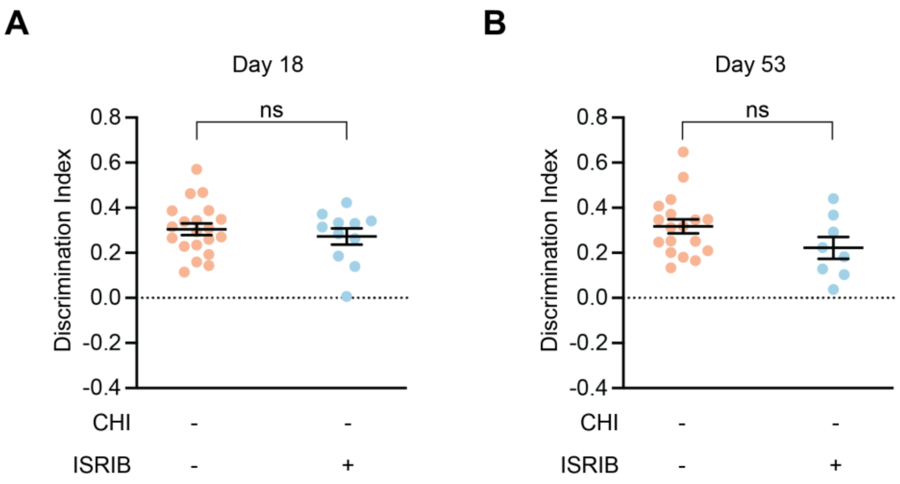

Supplemental Figure S7. ISRIB treatment does not affect sham control mice performance on novel object recognition task.

(A-B) The discrimination index at day 18 **(A)** or day 53 **(B)** for Sham ± ISRIB. For both **(A)** and **(B)**, sham mice that received ISRIB or Vehicle treatment showed no significant difference. **Statistics:** Analysis was done by Unpaired T-test (A-B). Data in (A) and (B) are means ± SEM. Sham ± ISRIB n = 31.

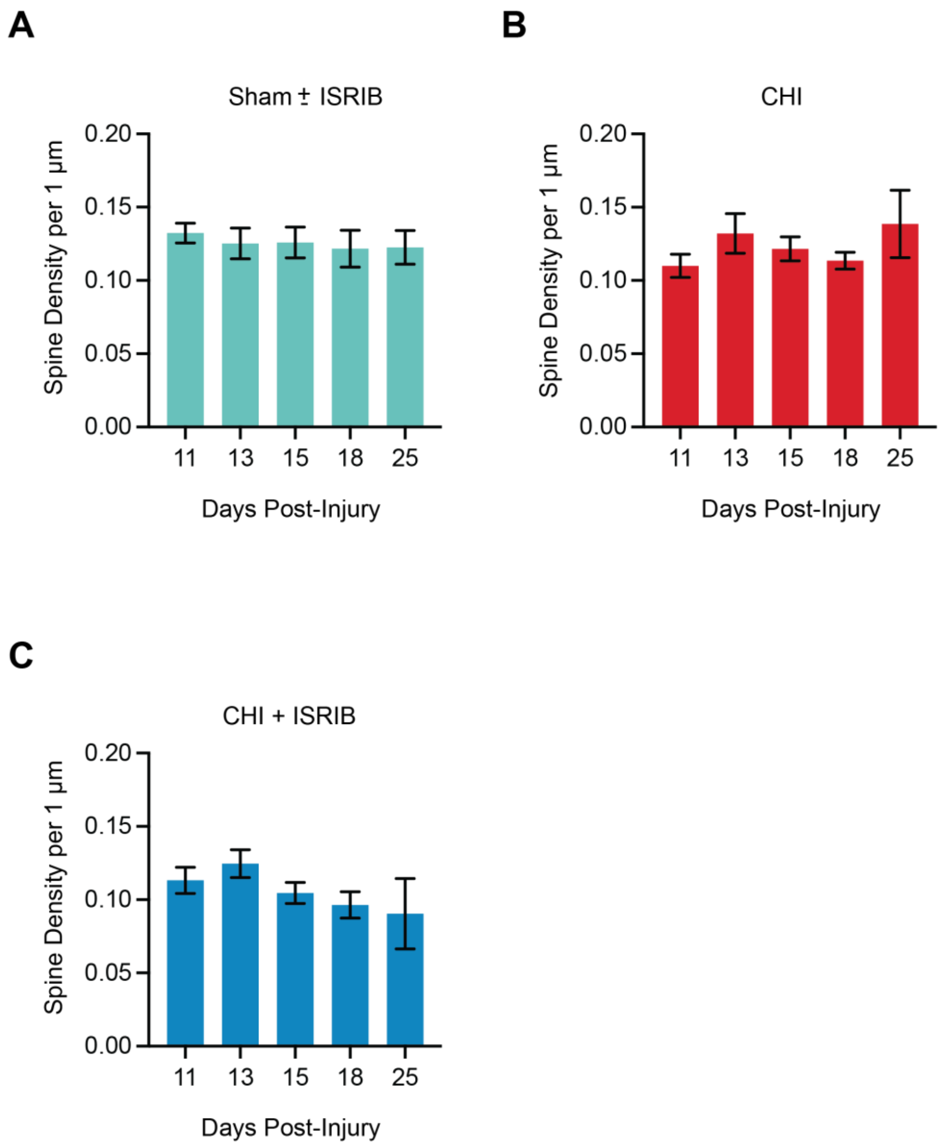

Supplemental Figure S8. Spine density per 1 µm for each group at days 11-25 post-injury.

(A-C) Spine density was quantified as the number of spines per micrometer of dendritic length from the longitudinal data for Sham ± ISRIB (A), CHI (B), or CHI + ISRIB (C). No significant differences in spine density were measured among groups. Sham mice that received ISRIB or Vehicle treatment showed no significant difference and were pooled and graphed as Sham ± ISRIB. Statistics were calculated between the experimental groups and the pooled Sham ± ISRIB treatment. **Statistics:** Analysis was done by two-way repeated measures ANOVA comparing all groups in (A-C). All data are means ± SEM. Sham ± ISRIB n = 13; CHI n = 12; CHI + ISRIB n = 11.

Supplemental Table S1. Detailed summary of the data sampling for dendritic spine analysis.

	Sham+Vehicle	Sham+ISRIB	CHI+Vehicle	CHI+ISRIB
Number of animals	6	7	12	11
Total number of dendrites	48	40	52	57
Total dendritic length distance (μm)	3912.6	3500.0	4042.2	4284.7
Mean dendritic segment length (μm)	78.4 ± 4	84.9 ± 9	76.8 ± 8	78.4 ± 8
